# Women's input and decision-making in agriculture are associated with diet quality in rural Tanzania

**DOI:** 10.3389/fpubh.2023.1215462

**Published:** 2023-12-06

**Authors:** Isabel Madzorera, Lilia Bliznashka, Mia M. Blakstad, Alexandra L. Bellows, Chelsey R. Canavan, Dominic Mosha, Sabri Bromage, Ramadhani A. Noor, Patrick Webb, Shibani Ghosh, Joyce Ludovick Kinabo, Honorati Masanja, Wafaie W. Fawzi

**Affiliations:** ^1^Division of Community Health Sciences, School of Public Health, University of California, Berkeley, Berkeley, CA, United States; ^2^Department of Global Health and Population, Harvard T. H. Chan School of Public Health, Boston, MA, United States; ^3^Nutrition, Diets, and Health Unit, International Food Policy Research Institute, Washington, DC, United States; ^4^Department of International Health (Human Nutrition), Johns Hopkins Bloomberg School of Public Health, Baltimore, MD, United States; ^5^Ifakara Health Institute, Dar es Salaam, Tanzania; ^6^Institute of Nutrition, Mahidol University, Salaya, Thailand; ^7^Department of Nutrition, Harvard T. H. Chan School of Public Health, Boston, MA, United States; ^8^Friedman School of Nutrition Science and Policy, Tufts University, Boston, MA, United States; ^9^Department of Food Science Technology, Nutrition and Consumer Sciences, Sokoine University of Agriculture, Morogoro, Tanzania; ^10^Department of Epidemiology, Harvard T. H. Chan School of Public Health, Boston, MA, United States

**Keywords:** women's empowerment, diet quality, decision-making, women, women's participation, agriculture, sub-Saharan Africa

## Abstract

**Background:**

Women's empowerment is one critical pathway through which agriculture can impact women's nutrition; however, empirical evidence is still limited. We evaluated the associations of women's participation, input, and decision-making in key agricultural and household activities with women's diet quality.

**Methods:**

We analyzed data from a cross-sectional study of 870 women engaged in homestead agriculture. We used food frequency questionnaires to assess women's diets and computed women's diet quality using the Prime Diet Quality Score (PDQS) (range 0–42), which captures healthy and unhealthy foods. We evaluated women's decision-making in 8 activities, food crop farming, cash crop farming, livestock raising, non-farm economic activities, wage/salary employment, fishing, major household expenditures, and minor household expenditures. Generalized estimating equations (GEE) linear models were used to evaluate associations between (a) women's participation, (b) decision-making, (c) adequate input, (d) adequate extent of independence in decision-making in agriculture, and (e) adequate input in use of agricultural income with their PDQS. Adequate input was defined as input into some, most or all decisions compared to input into few decisions or none. Adequate extent of independence was defined as input to a medium or high extent compared to input to a small extent or none.

**Findings:**

Median PDQS was 19 (IQR: 16–21). Women's adequate input in decision-making on wage and salary employment (estimate: 4.19, 95% CI: 2.80, 5.57) and minor expenditures were associated with higher PDQS vs. inadequate input. Women with independence in decision-making on livestock production (estimate: 0.97, 95% CI: 0.05, 1.90) and minor household expenditures, and women with adequate decision-making in the use of income from wages/salaries (estimate: 3.16, 95% CI: 2.44, 3.87) had higher PDQS. Participation in agricultural activities was positively associated with PDQS.

**Conclusions:**

Women's participation and input in decision-making in wage and salary employment, livestock production, and minor household expenditures were strongly associated with the consumption of better-quality diets. Women participating in multiple farm activities were also likely to have better diet quality. This study adds to the growing evidence on the pathways through which women's empowerment may influence women's nutrition in rural Tanzania.

## Background

Women's diet quality influences their nutrition and health, as well as that of their offspring. In many parts of Africa and Asia, women experience sub-optimal micronutrient intake and chronic energy deficiency because their diets consist primarily of staples with limited intake of nutrient-rich animal-source foods, vegetables and fruits (1). Recent transformations of global and local food systems have also contributed to the problems by increasing the availability of refined, processed, fast and unhealthy foods for women even in rural settings of low- and middle-income countries (LMICs) (2). It is not surprising that women in LMICs are increasingly facing a triple burden of malnutrition (persistent undernutrition, micronutrient deficiencies, and increasing overweight and obesity) (2, 3). In Tanzania, 10% of the women are underweight, 45% are anemic and overweight and obesity affect 18 and 10% of the women, respectively (4). Consumption of quality diets can address these key nutrition challenges among Tanzanian women.

For women involved in agriculture in Tanzania, access to resources and decision-making in agriculture may be important for their diets and nutrition. Literature suggests that for women involved in agriculture, empowerment may entail increasing their decision-making authority in relation to agricultural resources, management and production, and income (5). In Sub-Saharan Africa, women make up at least 40% of the agricultural labor, yet they face severe constraints, including a lack of access to inputs and other production resources required to meet their production potential (6). When women are not empowered, their access to the physical and human resources required to adopt optimal nutrition practices for their improved health is limited (7). Women tend to spend a greater proportion of household income on food purchases compared to men (5), and when they have more input in making decisions and have nutritional knowledge, they may act on that knowledge to provide higher quality diets for themselves and their families. Therefore, understanding the role of women's empowerment, input and decision-making in agriculture, and how that affects nutrition is important (8).

The empirical evidence linking women's empowerment with their nutrition outcomes has been increasing. Women's empowerment has been positively associated with women's diets and lower risk of maternal undernutrition (9–13). Constructs of women's empowerment such as empowerment in credit decisions, group membership and control over income have also been associated with women's dietary diversity (9, 10). However, most prior studies were cross-sectional and evaluated women's dietary diversity rather than overall diet quality. Dietary diversity represents only one dimension of diet quality, that is micronutrient adequacy (14), and does not consider the consumption of unhealthy foods and nutrients which have been increasing in LMICs and are linked to increased risk of non-communicable diseases (NCDs) such as diabetes and cardiovascular disease (2, 15–18). In a previous study, we assessed women's diet quality (including unhealthy foods) in urban Dar es Salaam using a novel tool, the Prime Diet Quality Score (PDQS) and found that women's diet quality was poor and associated with risk of low birth weight and preterm births (19). There is a need to further evaluate the risk factors for poor maternal diet quality in rural Tanzania, including the role of women's empowerment.

We believe that women's agency (including processes of decision-making) is an important component of women's empowerment and an important determinant of their nutrition. It reflects women's decision-making in intra-household resource allocation activities related to dietary intake, and their ability to act on their nutrition knowledge (20). Women also often have different preferences for allocating food and non-food resources compared to men, with benefits to their nutrition and health (5).

In this study, we evaluated associations of constructs of women's empowerment with their diet quality in rural Tanzania. Specifically, we evaluated the associations of (a) women's input in agriculture and household decision-making, (b) the extent to which women could provide input if they need to, and (c) women's decision-making on the use of income from agricultural activities, with women's diet quality, as measured by the PDQS in rural Tanzania. To our knowledge, no studies have evaluated the relationship between women's decision-making in agriculture and dietary quality.

## Materials and methods

### Study population

This study sample included participants enrolled in the Health and Demographic Surveillance System (HDSS) in Rufiji district, Tanzania. We used data from the Homestead Agriculture and Nutrition (HANU) project, a pair-matched cluster-randomized trial that sampled from participants from the HDSS and evaluated the effect of homestead gardening and nutrition and health education on women's diets. Details of the intervention have been published elsewhere (21, 22). The Rufiji HDSS is a repeated study and surveillance system that was established in 1998. It tracks households over time and collects data on the structural, behavioral, socio-economic and biological drivers of health and their impacts on the community (23). It generates information on longitudinal health and demographic indicators to guide national policy and decision-making (23).

Briefly, the HANU study selected 36 villages that were close to sources of water bodies for home gardening and to food markets as eligible for the study out of the 94 villages that are part of the Rufiji HDSS (24). From the 36 eligible villages, the study randomly selected and matched 10 eligible villages (5 pairs) from the HDSS and allocated them to the intervention (homestead gardening) or control groups (24). The study then enrolled 1,006 women of reproductive age (18–49 years), with at least one child aged 6–36 months and with access to pieces of land for vegetable production from the selected villages. The intervention was a homestead gardening intervention that provided seeds and training by agricultural extension officers to support the production of vegetables such as amaranthus, okra, and spinach by the study households, as well as nutrition education to promote the consumption of produced foods.

The study was implemented from August 2016 to December 2019 (25). The baseline conducted between August and October 2016 and a midline assessment from August-October 2017, 12 months after the intervention started. We used data from 880 women who participated in the midline assessment of the study for this analysis. We used data from the midline study only as it had allowed women to access the intervention that could impact the quality of their diets.

Research assistants collected data on household and women's socio-demographic characteristics, asset ownership and dietary intake. Maternal anthropometric measures of weight and height were also made. We collected data on household agricultural production and women's participation and decision-making in key agriculture activities. The interviews regarding women's decision-making were conducted where other members of the household or community could not overhear or contribute answers.

### Exposure variables

#### Women's empowerment

Conceptual frameworks have shown complexities in relationships in food systems and nutrition, and have posited that women's empowerment is a key component of impact pathways through which food systems could affect women's nutrition (26–29). Women's empowerment (WE), however, is a multi-dimensional and complex construct that is difficult to define and measure. It has been defined as a process through which women who have been denied the ability to make strategic choices acquire the ability to do so, and it encompasses access, the capability to make choices, and control over resources (30, 31). It is from this construct that the empowerment of women in agriculture metrics have been developed.

There are multiple ways to measure WE but only the Women's Empowerment in Agriculture Index (WEAI) has been widely applied to agricultural contexts (32). The WEAI assesses the empowerment, agency and inclusion of women in agriculture, and focuses on 5 domains (1) decisions about agricultural production, (2) access to and decision-making power on productive resources, (3) control of the use of income, (4) leadership in the community, and (5) time allocation (32). However, the time demands for the WEAI are high and it is more suited to producing country-level estimates (33). A project-level WEIA (pro-WEIA) (33) has also been developed, however, it is also time intensive. Therefore, a gap exists for simpler tools for assessing WE that can be easily incorporated into agriculture programs to track progress.

We assessed women's participation and decision-making in agriculture and household activities using select questions adopted from the WEAI questionnaire [4]. Women's empowerment was determined based on their participation in the following agricultural and household activities: (1) food crop farming: crops grown primarily for household food consumption; (2) cash crop farming: crops grown primarily for sale in the market; (3) livestock raising; (4) non-farm economic activities: e.g., running a small business, self-employment, buy and sell businesses; (5) wage and salary employment: work that is paid for in cash or in-kind, including agriculture and other wage work; (6) fishing or fishpond culture; (7) major household expenditures (e.g., purchases of bicycles, land, and small motorcycles); and, (8) minor household expenditures (e.g., purchasing food for daily consumption or other household needs).

For each activity, women were asked five questions which we used to define our exposure variables. Supplementary Table 1 shows the questionnaire used for the assessment, which is an excerpt from the WEIA questionnaire. We selected several questions from the WEIA around women's decision-making and the extent of their input (including their scoring guidelines) in key agriculture related activities. We assessed the following:

(a) *Participation in agricultural activity*: Women were asked if they participated (alone or with others) in household activities in the past 12 months. We developed a binary score for women's participation in agriculture and household activities (yes/no).
(b) *Decision-making regarding activity*: We asked women if they reported participating in selected activities, did they make decisions around these activities individually or jointly with others in the households. We calculated a binary score for women's participation in decision-making (yes/no).
(c) *Adequate input in decision-making in agriculture activities:* If they reported participating in the selected activities, we also asked women how much input they provided in decision-making in the 8 agricultural and household activities. We classified responses as “Yes” when they had input into some, most, or all decisions, or “No” if they had no input or input into a few decisions for each activity (adequate women's input, binary variable, yes/no).
(d) *Adequate extent of independence in decision-making in agriculture activities:* We asked women about the extent to which they could (hypothetically) provide input in decision-making in the eight agricultural and household activities. We classified responses as “Yes” when they had input to a medium or high extent, or “No” if they had no input or input to a small extent (adequate extent of independence in decision-making, binary variable, yes/no).
(e) *Adequate input in use of income from agriculture activities:* Finally, we asked women if they participated in decision-making on the use of income from agricultural activities, including for household expenditures. We defined responses as “Yes” when they provided input into some, most, or all decisions, or “No” when they provided no input or input into a few decisions (adequate decision-making on the use of income from agriculture, binary variable, yes/no).

We also summed up the total number of activities that women participated in (range 0–8), to calculate a participation score. We classified women's participation scores into tertiles.

**Figure 1:**
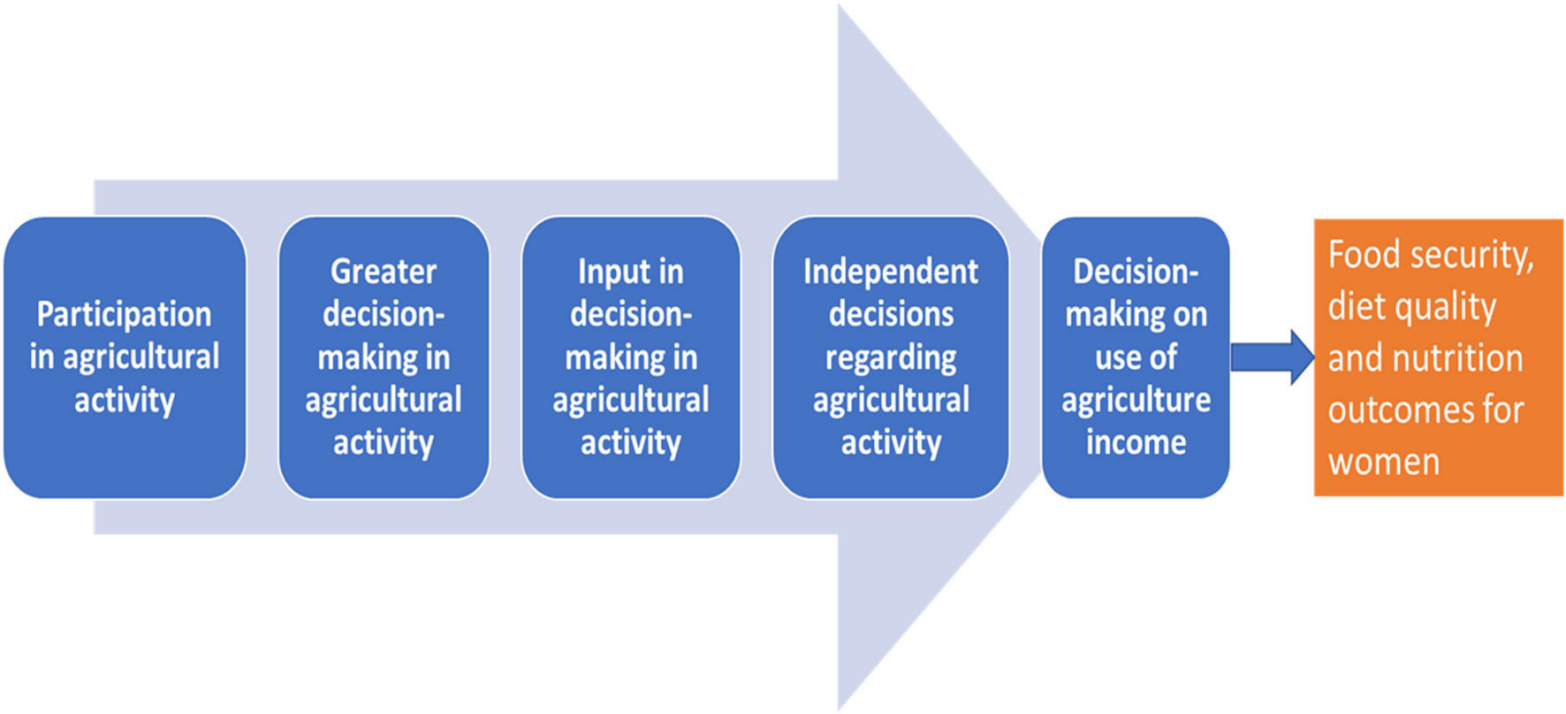
Theory of change.

Figure 1 shows our theory of change on how these factors could influence women's diets and nutrition outcomes. We hypothesized that different forms of women's participation in agriculture would impact women's diets and nutrition through their influence on their food security. We also hypothesized that women's participation in agricultural activities alone may not be sufficient to optimize their dietary intake. We posit that as we move from participation to decision-making this represents greater empowerment, as does independence in making decisions (if required) and control over financial resources; and as these improve, the impacts on women's diets and nutrition would be greater. Further, we also suggest that participation in some activities may be more important than others for women's diets.

### Outcome variable

Women's dietary intake was assessed using a food frequency questionnaire (FFQ) which was administered by research assistants. A previously validated FFQ composed of a list of 79 common local foods was used (34). Women were asked to recall how often they consumed foods over the previous month. Frequency of food consumption was recorded as: 0 times in a month, 1–3 times per month, 1 time per week, 2–4 times per week, 5–6 times per week, 1 time per day, 2–3 times per day, 4–5 times per day, and 6 or more times per day.

Women's diet quality was assessed using the Prime Diet Quality Score (PDQS). The PDQS has been used as a measure of diet quality in urban Tanzania where it was shown to be associated with low birth weight and preterm births (19), and has been shown to predict gestational diabetes, hypertension diabetes, and coronary heart disease among women in high-income contexts (16–18). The PDQS is a measure of overall diet quality, capturing the diversity of healthy and unhealthy food consumption (15).

We classified foods consumed by women in the previous month into 21 food groups for the PDQS as follows, 14 healthy food groups (dark green leafy vegetables; other vitamin A rich vegetables; cruciferous vegetables; other vegetables; whole citrus fruits; other fruits; fish; poultry; legumes; nuts; low-fat dairy; whole grains; eggs; and, liquid vegetable oils) and 7 unhealthy food group (red meat; processed meats; refined grains and baked goods; sugar-sweetened beverages; desserts and ice cream; fried foods obtained away from home and potatoes) based on criteria determined by previous studies (16, 17). We included roots and tubers in the potatoes group, and maize flour-based products including ugali as refined grains. Based on the frequency of consumption, we assigned points for healthy foods as follows: 0–1 serving/week (0 points), 2–3 servings/week (1 point) and ≥4 servings/week (2 points). For unhealthy food groups we assigned points as: 0–1 serving/week (2 points), 2–3 servings/week (1 point) and ≥4 servings/week (0 points) (16).

Processed meat intake, low-fat dairy, liquid vegetable oil consumption were not measured in the study, therefore all women were assigned low intake for these groups, that is 2 points for processed meats and 0 points for the others. We summed the healthy and unhealthy foods scores to compute the overall PDQS for each woman (range 0–42).

#### Ethics

Written informed consent was obtained from all enrolled women. Ethical approval for the study was provided by the Ifakara Health Institute (IHI) independent research board, the Medical Research Council Committee of the National Institutes of Medical Research (NIMR) in Tanzania (NIMR/HQ/R. 8 a/Vol. IX/2262), and the Harvard T. H. Chan School of Public Health institutional review board. The trial was registered with clinicaltrials.gov (ClinicalTrials.gov NCT03311698).

### Statistical analysis

We described the background characteristics of the women included in our study. Chi square *p*-values were reported for categorical/binary variables and the Wilcoxon test for continuous variables was used to describe differences in background characteristics by tertile of participation in agricultural activities. We described the frequency of consumption of the PDQS food groups, and women's participation and input into agriculture decisions, the extent of their independent input in agriculture decisions and their decision-making in use of income from agriculture using counts and frequencies and means and standard deviations.

We used generalized estimating equation (GEE) linear models with exchangeable correlation and adjusting for clustering by village pair to evaluate univariate associations of (a) women's participation in agriculture activities, (b) women's decision-making, (c) women's adequate input into decision-making in agriculture, (d) women's adequate extent of independence in decision-making in agriculture activities, and (e) women's decision-making in the use of agricultural income with women's diet quality (PDQS).

We also estimated adjusted models, selecting confounders based on univariate associations between potential confounders and PDQS at levels of *p* < 0.20. We adjusted for participation in the homestead gardening intervention (intervention vs. control). We considered the following potential confounders: women's age (18–24, 25–34, 35–49 years), women's education (none, primary, secondary, or higher), marital status (married/not married), women's body mass index (underweight: BMI < 18.5, normal weight: BMI 18.5–24.9, and overweight and obesity: BMI ≥ 25 kg/m^2^), parity (0–1, ≥2), family size, wealth index (calculated using factor analysis of nine items for asset ownership and housing quality), land size (acres), weekly income (log), food expenditure (log), and distance to market (km). Multivariable associations were considered statistically significant at *p* < 0.05. We used the missing indicator method to account for missing covariate data (35). Analysis was conducted using SAS 9.4 program.

## Results

We analyzed data from 870 women (excluding 10 women ≤18years or older than 50 years). The mean age of study women was 31(±8) years. At least 77% of the women were married, 34% had no primary school education, and 57% had only primary school education. On average, women reported participating in 3 (±2) agricultural activities out of a possible 8 activities. Women in the highest tertile of the agriculture participation score were older, and more likely to have more than 2 children (Table 1). They also lived further from markets. In addition, women participating in more agricultural activities had access to larger pieces of land (4.1 ± 3.9 vs. 2.8 ± 2.5 acres) and were more likely to have sold at least 1 food crop in the previous year (68.9% vs. 31.5%) compared to women participation in a few activities. However, they spent less money on food purchases compared to women in the lowest tertile. Women who reported participating in ≥3 agricultural activities (median) were more likely to be older, married, and less educated, compared to women who participated in 2 or fewer activities. Overweight and obesity were high affecting 24.3% and 13.1% of women, and underweight affected 6.8% of the women, respectively. Women's PDQS was low (median 19, IQR: 16–21, maximum score 42).

**Table 1:** Socio-economic and demographic characteristics of study women in Tanzania by participation in agricultural activities (*N* = 870).

	**Number of agricultural and household activities women participate in**		
	**Tertile 1 (0–1)**	**Tertile 2 (2, 3)**	**Tertile 3 (4–8)**
	***N*** = **203**	***N*** = **408**	***N*** = **257**
**Maternal age (years)**	29.8 ± 7.7	31.0 ± 7.5	33.5 ± 7.6^***^
18–24	65 (32.0)	97 (23.8)	38 (14.8)^***^
25–34	76 (37.4)	184 (45.1)	94 (36.6)^***^
35+	62 (30.5)	127 (31.1)	125 (48.6)^***^
**Marital Status**			
Married	151 (74.3)	303 (74.3)	218 (84.8)^*^
**Body mass index (BMI kg/m2)**			
Underweight (BMI < 18.5)	12 (5.9)	24 (5.9)	23 (9.1)
Normal weight (BMI 18.5–24.99)	113 (55.9)	236 (58.0)	132 (52.2)
Overweight (BMI 25–29.99)	54 (26.7)	93 (22.9)	62 (24.5)
Obese (BMI ≥ 30)	23 (11.4)	54 (13.3)	36 (14.2)
**Maternal education**			
None	52 (25.6)	145 (35.5)	96 (37.4)
Primary school	129 (63.6)	226 (55.4)	144 (56.0)
Secondary school or higher	22 (10.8)	37 (9.1)	17 (6.6)
**Paternal education**			
None	38 (23.8)	83 (27.0)	48 (21.7)
Primary school	93 (58.1)	182 (59.1)	146 (66.1)
Secondary school or higher	29 (18.1)	43 (14.0)	27 (12.2)
**Parity**			
1 child or none	56 (27.6)	86 (21.1)	24 (9.3)^***^
2 or more children	147 (72.4)	322 (78.9)	233 (90.7)^***^
**Family size**	6.5 ± 3.0	6.4 ± 2.5	6.7 ± 2.4
**Household wealth quintile**			
First (lowest)	32 (15.8)	81 (19.9)	60 (23.4)
Second	45 (22.2)	112 (27.5)	74 (28.8)
Third	29 (14.3)	51 (12.5)	29 (11.3)
Fourth	48 (23.7)	84 (20.6)	50 (19.5)
Fifth (highest)	49 (24.1)	80 (19.6)	44 (17.1)
**Household food expenditure (Tanzanian shillings)**	7,639 ± 4,076	7,297 ± 3,440	7,141 ± 5,137^***^
**HANU assignment**			
Treatment	87 (42.9)	205 (50.3)	155 (60.3)^**^
Control	116 (57.1)	203 (49.8)	102 (39.7)^**^
**Plot size (acres)**	2.8 ± 2.5	3.1 ± 3.8	4.1 ± 3.9^***^
**Livestock ownership, mean (**±**SD)**			
Chickens	9.7 ± 8.2	9.2 ± 15.2	9.3 ± 15.1
Goats	1.2 ± 4.6	1.2 ± 4.2	0.8 ± 2.7
**Sold at least 1 crop in the previous year**	64 (31.5)	201 (49.3)	177 (68.9)^***^
**Distance to market (km) Median (IQR)**	0.9 [0.6–1.3]	1.1 [0.8–1.5]	1.4 [0.9–5.7]^***^
**PDQS Median (IQR)**	19.0 [(16.0–21.0)]	19.0 [(17.0–21.0)]	19.0 [(16.0–21.0)]

* *p* < 0.05,

** *p* < 0.01,

*** *p* < 0.001. Values are mean ± SD, median [IQR], or frequency (percent); PDQS, Prime Diet Quality Score. Chi-square *p*-values were reported for categorical/binary variables and the Wilcoxon test was for continuous variables. Exchange rate in November 2016: 2,200 Tanzanian shillings per $US1.

Among the healthy food groups, women consumed other vegetables (97.1%), fish (89.4%), legumes (81.6%), and dark green vegetables (62.3%; Table 2) at least 4 times each week. The most frequently consumed unhealthy food groups (where consumption in moderation is preferred) were refined grains (100%) and potatoes, roots, and tubers (82.7%) which were consumed at least 4 times per week on average. Median PDQS was 19 (IQR: 17–21).

**Table 2:** Proportion of women reporting consumption of PDQS food groups in rural Tanzania.

**Healthy foods**			
	**0–1 serving/wk**	**2–3 servings/wk**	≥**4 servings/wk**
**Servings and points**	**(0 points)**	**(1 point)**	**(2 points)**
Cruciferous vegetables	706 (81.3)	133 (15.3)	29 (3.3)
Dark leafy green vegetables	137 (15.8)	190 (21.9)	541 (62.3)
Eggs	839 (96.7)	26 (3.0)	3 (0.4)
Fish	5 (0.6)	87 (10.0)	776 (89.4)
Legumes	46 (5.3)	114 (13.1)	708 (81.6)
Liquid vegetable oils^d^	870 (100)	0 (0)	0 (0)
Low-fat dairy^d^	870 (100)	0 (0)	0 (0)
Nuts	793 (91.4)	61 (7.0)	14 (1.6)
Other vegetables	5 (0.6)	20 (2.3)	853 (97.1)
Other vitamin A-rich vegetables (incl. carrots)	469 (54.0)	208 (24.0)	191 (22.0)
Other whole fruits	397 (45.7)	244 (28.1)	227 (26.2)
Poultry	818 (94.2)	40 (4.6)	10 (1.2)
Whole citrus fruits	324 (37.3)	295 (34.0)	249 (28.7)
Whole grains	532 (61.3)	209 (24.1)	127 (14.6)
**Unhealthy foods**			
	**0–1 serving/wk**	**2–3 servings/wk**	≥**4 servings/wk**
**Servings and points**	**(2 points)**	**(1 point)**	**(0 points)**
Desserts and ice cream^a^	190 (21.9)	307 (35.4)	371 (42.7)
Fried foods away from home	830 (95.6)	33 (3.8)	5 (0.6)
Potatoes^b^	29 (3.3)	121 (13.9)	718 (82.7)
Processed meats^d^	870 (100)	0 (0)	0 (0)
Red meats	777 (89.5)	83 (9.6)	8 (0.9)
Refined grains, baked goods^c^	0 (0)	0 (0)	868 (100)
Sugar-sweetened beverages	566 (65.2)	193 (22.2)	109 (12.6)

^*^p < 0.05,

^**^p < 0.01,

^***^p < 0.001, wk, week. Values are mean ± SD, median [IQR], or frequency (percent); PDQS, Prime Diet Quality Score.

a The desserts and ice cream group includes cakes, doughnuts, rice cake, honey, and ice cream.

b A roots and tubers group was used in place of a “potatoes” group in the original score. This category includes potatoes, sweet potatoes, and taro.

c Maize flour-based products are classified as refined grains.

d Processed meat intake, low-fat dairy, liquid vegetable oil, and were not measured in the study, therefore all women were assigned low intake for these groups, that is 2 points for processed meats and 0 points for the others.

**Figure 2:**
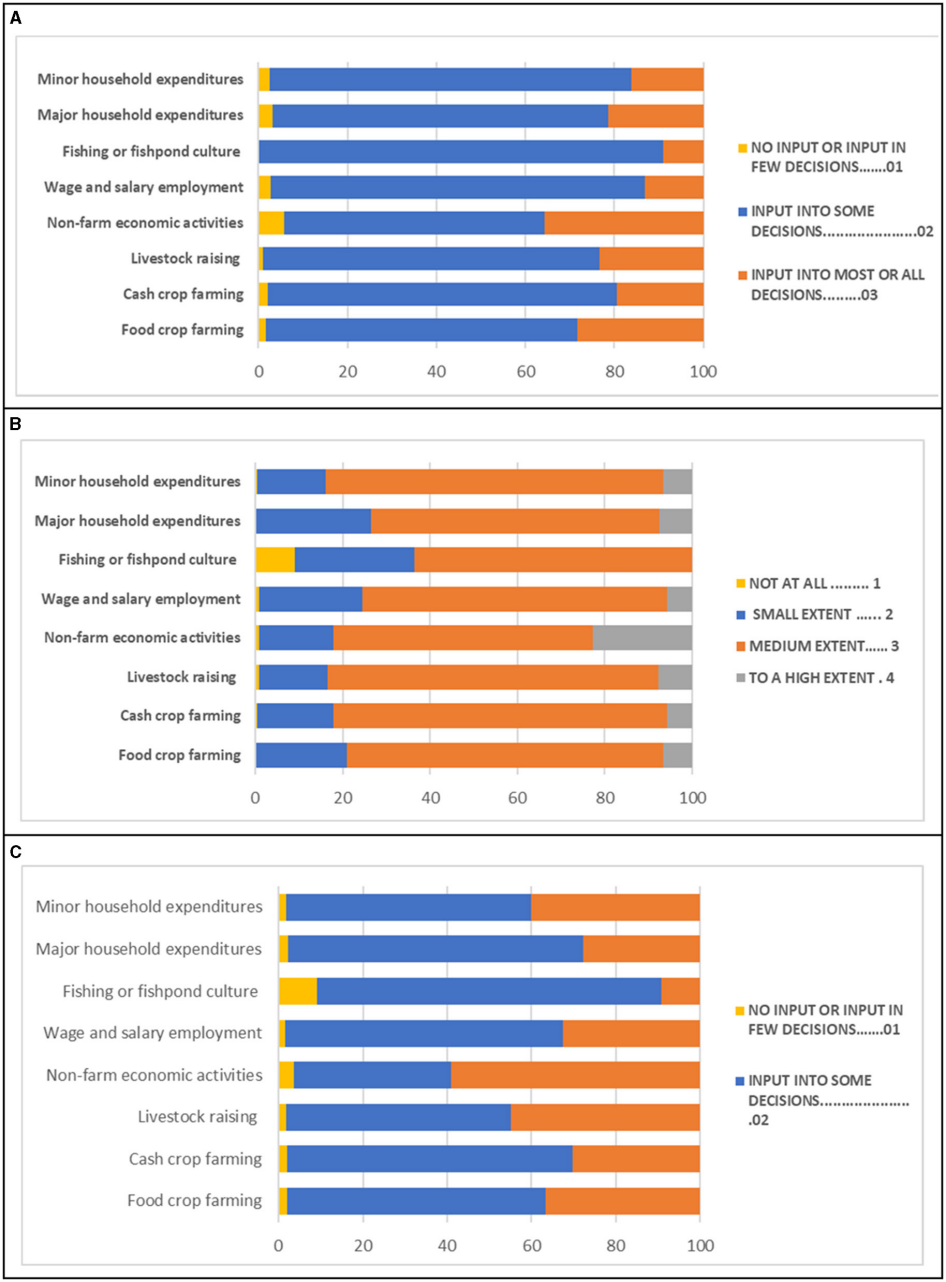
Prevalence of **(A)** women's input in decision-making in agricultural activities **(B)** women's extent of independence in decision-making in agricultural activities, and **(C)** women's input in decision-making on the use of income from agricultural activities and for household expenditures in rural Tanzania.

Most of the women in our study reported participating in food crop farming (77.7%) and minor household expenditures (69.4%) (Supplementary Table 2). Women's participation in the other activities we assessed was low (<36%). Women's reported participation was lowest for fishing (1.4%), wage and salaried employment (15.7%), and livestock raising (18.7%). When we only considered women who reported participating in the activity, more than 80% reported making decisions in minor household expenditures, non-farm economic activities, cash crop farming and livestock raising (Supplementary Table 2). Figure 2 shows women's input in decision-making in agriculture and household activities. On average, among women reporting participating in each activity, most reported providing input into some decisions (Figure 2A), the ability (hypothetical) to participate in decision-making to a medium extent (Figure 2B), and participating in decision-making and providing input into some decisions on the use of income (Figure 2C).

In adjusted models, women's participation in non-farm economic activities (estimate: 0.62, 95% CI: 0.14, 1.10) and wage and salaried employment (estimate: 1.15, 95% CI: 0.25, 2.04) were positively associated with PDQS (Table 3). Women who reported participating in decision-making alone or jointly with other household members in food crop farming (estimate: 0.67, 95% CI: 0.10, 1.23), cash crop farming (estimate: 1.05, 95% CI: 0.16, 1.95), livestock farming (estimate: 1.33, 95% CI: 0.95, 1.72), wage and salaried employment (estimate: 0.87, 95% CI: 0.64, 1.10), major (estimate: 0.44, 95% CI: 0.17, 0.72) and minor household expenditures (estimate: 0.96, 95% CI: 0.50, 1.43) had higher PDQS.

**Table 3:** Associations of women's participation in agricultural and household activities and decisions-making on household life with women's PDQS.

**Activity**	**Woman participates in activity** ^**a**^			**Woman contributes to decision-making regarding activity** ^**b**^		
	***n/N***	**Univariate estimate**	**Adjusted estimate** ^c^	***n/N***	**Univariate estimate**	**Adjusted estimate** ^c^
Food crop farming	674/868	0.15 (0.03, 0.28)^*^	0.11 (−0.03, 0.24)	528/672	−0.11 (−1.03, 0.81)	0.67 (0.10,1.23)^*^
Cash crop farming	308/868	0.29 (−0.14, 0.72)	0.11 (−0.27, 0.48)	250/306	0.18 (−0.60, 0.96)	1.05 (0.16, 1.95)^*^
Livestock raising	162/868	0.40 (0.06, 0.74)^*^	0.61 (−0.33, 1.55)	132/162	0.18 (−0.31, 0.67)	1.33 (0.95, 1.72)^***^
Non-farm economic activities	252/868	0.60 (0.22, 0.98)^**^	0.62 (0.14, 1.10)^*^	212/252	0.49 (0.13, 0.84)^*^	0.92 (−0.37, 2.21)
Wage and salary employment	136/868	0.87 (0.43, 1.32)^***^	1.15 (0.25, 2.04)^*^	103/136	0.46 (−0.17, 1.10)	0.87 (0.64, 1.10)^***^
Major household expenditures	190/868	0.86 (0.55, 1.17)^***^	0.46 (−0.05, 0.97)	130/190	0.50 (−0.58, 1.58)	0.44 (0.17, 0.72)^**^
Minor household expenditures	602/868	0.31 (−0.33, 0.95)	−0.27 (−1.34, 0.80)	508/602	1.43 (1.04, 1.82)^***^	0.96 (0.50, 1.43)^***^

PDQS, Prime Diet Quality Score,

* *p* < 0.05,

** *p* < 0.01,

*** *p* < 0.001. Generalized estimating equation (GEE) linear models with exchangeable correlation and adjusting for clustering by village pair were used to evaluate the association of women's empowerment with women's diet quality.

a Woman decision-making in activity alone or jointly with other household members—(see Question 1 of questionnaire in Supplementary Table 1).

b See Question 2 of questionnaire in Supplementary Table 1).

c Adjusted models control for treatment assignment (treatment/control), woman's age (18–24 years, 25–34 years, 35–49 years), woman's education (none, primary, secondary and higher), parity (0–1, 2+), wealth index (quintiles), land size (acres), livestock diversity, weekly income (log), household food expenditure (log), and distance to market. Models for fishing are not shown due to non-convergence of models.

With respect to adequate input in decision-making, we found that women with adequate input in decision-making on wage and salary employment had 4.19 (95% CI: 2.80, 5.57) points higher PDQS compared to women with inadequate input (Table 4). In addition, women with adequate input in minor expenditures had 1.12 (95% CI: 0.78, 1.45) points higher PDQS compared to women without adequate input. However, women reporting adequate input in major household expenditures had lower PDQS (estimate −1.25, 95% CI: −2.39, −0.11), compared to women without adequate input.

**Table 4:** Associations of adequate input in decision-making, extent of decision-making, and adequate input in use of agricultural income with women's PDQS.

		**Woman has substantial input in decision-making related to activity**^**a**^^,^ ^**d**^		**Woman can make her own personal decisions regarding activity**^**b**^^,^ ^**d**^		**Woman has substantial decision-making on use of income generated from activity**^**c**^^,^ ^**d**^	
**Activity**	***n/N***	**Univariate estimate**	**Adjusted estimate**	**Univariate estimate**	**Adjusted estimate**	**Univariate estimate**	**Adjusted estimate**
Food crop farming	674/868	−0.06 (−0.62, 0.51)	−0.30 (−1.37, 0.77)	0.26 (−0.61, 1.13)	0.65 (−0.07, 1.37)	0.16 (−0.51, 0.83)	−0.14 (−0.72, 0.45)
Cash crop farming	308/868	1.02 (0.25, 1.79)^*^	0.57 (−1.18, 2.33)	−0.49 (−1.05, 0.07)	−0.87 (−1.88, 0.15)	0.65 (−0.49, 1.79)	0.96 (−1.27, 3.20)
Livestock raising	162/868	1.75 (1.40, 2.09)^***^	0.11 (−1.17, 1.39)	−0.54 (−2.14, 1.06)	0.97 (0.05, 1.90)^*^	0.03 (−1.63, 1.69)	1.14 (−0.76, 3.05)
Non-farm economic activities	252/868	0.68 (0.50, 0.87)^***^	1.08 (−0.04, 2.20)	0.37 (−0.56, 1.31)	1.01 (−1.54, 3.56)	0.37 (−0.50, 1.24)	0.72 (−0.53, 1.97)
Wage and salary employment	136/868	2.66 (2.65, 2.67)^***^	4.19 (2.80, 5.57)^***^	−0.22 (−1.94, 1.49)	−0.54 (−2.57, 1.49)	2.63 (2.62, 2.64)^**^	3.16 (2.44, 3.87)^***^
Major household expenditures	190/868	0.79 (0.50, 1.07)^***^	−1.25 (−2.39, −0.11)^*^	0.97 (0.00, 1.95)^*^	0.80 (−0.15, 1.75)	1.56 (1.14, 1.98)^**^	0.81 (−0.81, 2.43)
Minor household expenditures	602/868	0.15 (−0.08, 0.37)	1.12 (0.78, 1.45)^**^	1.20 (0.53, 1.87)^**^	1.35 (0.54, 2.15)^**^	−0.15 (−1.99, 1.69)	0.93 (−0.85, 2.71)

PDQS, Prime Diet Quality Score;

* *p* < 0.05,

** *p* < 0.01,

*** *p* < 0.001. Generalized estimating equation (GEE) models with exchangeable correlation and adjusting for clustering by village pair were used to evaluate the association of women's empowerment with diet quality.

a Binary variables for adequate input in decision-making used. Adequate input is defined as input into some, most or all decisions, compared to no input or input into few decisions. A binary variable is used (Y/N) (see Question 3 of questionnaire in Supplementary Table 1).

b Binary variables for adequate extent of independence in decision-making in agriculture activities used. Adequate extent of decision-making is defined as input to a medium or high extent, compared to no input or input to a small extent. A binary variable is used (Y/N) (see Question 4 of questionnaire in Supplementary Table 1).

c Binary variables for adequate decision-making on use of income used. Adequate decision-making on use of income from agriculture is defined as input into some, most or all decisions compared to no input or input into few decisions. A binary variable is used (Y/N) (see Question 5 of questionnaire in Supplementary Table 1).

d Adjusted models control for treatment assignment (intervention vs. control), woman's age (18–24 years, 25–34 years, 35–49 years), women's education (none, primary, secondary, and higher), parity (0–2, 3+), wealth index (quintiles), land size (acres), weekly income (log), food expenditure (log), and distance to market. Model for livestock excludes food expenditure to enable convergence of model.

^e^ Models for fish farming are excluded due to non-convergence.

Women with adequate independence in decision-making in livestock production had 0.97 (95% CI: 0.05, 1.90) points higher PDQS and women with adequate independence in decision-making in minor household expenditures had 1.35 (95% CI: 0.54, 2.15) points higher PDQS compared to those with inadequate independence. Further, women's adequate decision-making in the use of income from wage and salary employment was associated with a 3.16-point higher PDQS (95% CI: 2.44, 3.87) in adjusted models. Finally, women who participated in more agricultural activities had higher PDQS (tertile 3 vs. 1: estimate: 0.78, 95% CI: 0.38, 1.18) in adjusted models (Table 5).

**Table 5:** Associations of women's participation in agricultural and household activities with women's PDQS.

	**Women's participation in agriculture score** ^**a**^			
	**Tertile 1 (0–1)**	**Tertile 2 (2, 3)**	**Tertile 3 (4–8)**	
	***N*** = **203**	***N*** = **408**	***N*** = **257**	**P for trend**
PDQS, Mean (SD)	18.51 ± 2.78	18.86 ± 2.68	18.93 ± 2.98	
Univariate	ref	0.57 (0.19, 0.94)^*^	1.16 (0.72, 1.59)^***^	< 0.001
Multivariate^b^	ref	0.35 (−0.47, 1.17)	0.78 (0.38, 1.18)^***^	< 0.001

PDQS, Prime Diet Quality Score,

* *p* < 0.05,

^**^ *p* < 0.01,

*** *p* < 0.001. Generalized estimating equation (GEE) linear models with exchangeable correlation, controlling for clustering by village pair, were used to evaluate the association of women's empowerment with maternal diet quality.

a Women's participation is calculated as the number of agricultural activities that women participate in. Classified as tertiles.

b Adjusted models control for treatment assignment (interventions vs. control), woman's age (18–24 years, 25–34 years, 35–49 years), woman's education (none, primary, secondary and higher), parity (0–2, 3+), wealth index (quintiles), land size (acres), weekly income (log), food expenditure (log), and distance to market.

## Discussion

We assessed the associations between women's participation in decision-making in agricultural and household activities and how they related to their diet quality in rural Tanzania. We found that women's participation in non-farm-economic activities and paid employment and their adequate input and decision-making in the use of income were associated with better diet quality. Women's decision-making and ability to make decisions regarding livestock rearing were also associated with better-quality diets. Finally, women who participated in more agricultural activities were more likely to have better diet quality.

Overall, our study's findings of associations between various dimensions of women's empowerment with diet quality are consistent with previous studies that have shown that women's empowerment is associated with women's diversity. A study in Uganda, Rwanda, Malawi, Zambia, and Mozambique found that women's greater input in production decisions was associated with the higher consumption of dairy products, fruits, and vitamin A-rich vegetables (36). Findings relating women's empowerment in economic domains and better diet quality are consistent with previous studies showing that women's empowerment in credit decisions in Ghana (10), and control over income in Nepal (9), were positively associated with women's dietary diversity. Studies have also shown associations between off-farm income and diets. In India, household access to non-farm income was associated with a lower likelihood of the household consuming poor quality diets (37), and in Nigeria, off-farm income improved household calorie supply (38).

Women's income-based empowerment likely works to enhance diet quality by increasing food purchases for her and her household and also influencing other non-food household expenditures (28). However, we lacked data on food purchases and were unable to empirically confirm this hypothesis. A systematic review found that women's share of household income-earned and share of land owned did not increase household food budgets (39). However, it is plausible that women with greater agency are either able to re-allocate the household budget (shifting it toward themselves rather than increasing it) or were able to start their own food budget (and thus purchase healthier foods for themselves). Future studies should collect detailed data on household and individual food purchases to help unpack the pathways through which income-based empowerment influences diet quality.

Previous studies have also shown associations between women's empowerment with women's diet diversity in Kenya, Ghana, Nepal, India, and Timor Leste (9, 10, 12, 13, 40, 41), as well as women's nutrition status in Benin (42).

We found that when women could make more independent decisions regarding livestock, their diet quality was better. This may be because women view decision-making power as being key to increasing their independence in livestock rearing (43). Livestock can play an important role in providing food and income, as well as increasing women's bargaining power (44). This is particularly true for small livestock that tend to be considered a women's responsibility in many LMICs. It is important to note that merely owning livestock is not sufficient for women to benefit, as they often have limited ownership rights pertaining to livestock, have restricted decision-making power and control over income, and are not often prioritized for access to livestock products (45). Therefore, control of resources related to livestock and decision-making around livestock may be crucial for women's diets, as well as, for their children (44). In our study, most households owned chickens and goats and we hypothesized that women may have benefitted from consuming poultry and other related products and from derived income from the sale of the livestock and their products.

The finding that participation in more agricultural activities was associated with better quality diets was contrary to some studies. One study in Bhutan suggested that the relationship between women's participation in decision-making in agricultural activities with household dietary diversity may be non-linear, with high levels of participation associated with less diverse household diets (46). Additionally, there is often the concern that participating in numerous agricultural activities increases women's time use burden, and takes away time for nutrition practices, to the detriment of women's nutrition (47). Further, additional energy expenditure by women for example during cultivating periods can adversely affect their nutrition and health (28). We hypothesized that participating in more agriculture activities can be a valuable coping strategy for the most vulnerable households as it helps diversify income and food sources. Higher and more stable income earned from these agricultural activities can then be used by women to procure more nutritious foods. Other studies have also indicated that resources and agency are important to ensure that women optimize their livelihoods and health outcomes (48). In our study, women who participated in more activities had access to larger plots of land and were more likely to sell crops in the previous season. Thus, diversification of agricultural activities could have benefitted women.

The strengths of this study include that it is one of the first studies to relate women's empowerment to the overall quality of diets consumed by women in Tanzania. The limitations of the study include the cross-sectional study design. Further, our paper seeks to investigate dimensions of women's participation in agriculture and how they relate to their diets. However, the limited number of papers on this topic and varying definitions of WE make comparability with other projects and approaches difficult. Finally, the questions that are used for the analysis (and their scoring) are part of the WEIA questionnaire which has been extensively validated (32). However, the independent evaluation of the sub-components has been limited thus far. However, in this study, we are doing construct validation of these sub-components against diet quality for women and this provides valuable information.

Despite these limitations, our findings have important research and policy implications. First, our findings are consistent with prior work using more comprehensive measures of women's empowerment. Thus, we believe that our approach of using simpler, proxy measures for women's empowerment, an approach also used by other scholars, has great promise (9, 10, 36, 49). Adopting simpler measures will make it easier for researchers and programs to evaluate and track progress in women's empowerment. Our findings that women's empowerment was associated with better quality diets suggest that interventions aiming to improve women's diets should explicitly promote women's empowerment. In Tanzania, interventions to promote women's empowerment in livestock rearing, participation in non-farm activities, and decision-making in the use of agricultural and non-agricultural income may be effective at improving women's diet quality. The study findings could be extrapolated to similar rural locations in Tanzania and other LMIC countries in Africa where similar conditions prevail such as limited access to water for vegetable gardening.

In conclusion, we assessed the associations between women's empowerment and diet quality in rural Tanzania. We found that women's input in decision-making about paid employment, women's decision-making in livestock production and minor household expenditures, and decision-making in the use of income from wages and salaried employment were associated with the consumption of better-quality diets. Increasing women's participation and decision-making in these key activities may be an important consideration for agriculture programs and policies that seek to improve women's diets and nutrition.

## Data availability statement

The datasets presented in this article are not readily available because of a data transfer agreement between the Harvard T. H. Chan School of Public Health and the Ifakara Health Institute. Requests to access the datasets should be directed to ghp@hsph.harvard.edu.

## Ethics statement

The studies involving humans were approved by Ethical approval for the study was provided by the Ifakara Health Institute (IHI) independent research board, the Medical Research Council Committee of the National Institutes of Medical Research (NIMR) in Tanzania (NIMR/HQ/R. 8 a/Vol. IX/2262), and the Harvard T. H. Chan School of Public Health institutional review board. The trial was registered with clinicaltrials.gov (ClinicalTrials.gov NCT03311698). The studies were conducted in accordance with the local legislation and institutional requirements. The participants provided their written informed consent to participate in this study.

## Author contributions

IM conceived and designed the study, analyzed the data, and drafted the manuscript. WF was the principal investigator for the parent study, conceived the study, designed the study, interpreted the data, and guided revisions of the manuscript. LB, MB, AB, CC, DM, SB, RN, PW, and SG designed the study, interpreted the data, and guided revisions of the draft manuscript. JK and HM were co-investigators for the study and participated in the study implementation, interpreted the data, and guided revisions of the manuscript. All authors read and approved the final manuscript.
