# Supplementary material for: Women's input and decision-making in agriculture are associated with diet quality in rural Tanzania
Source: Front Public Health. 2023 Dec 6;11:1215462. doi: 10.3389/fpubh.2023.1215462 (PMC10731380; doi:10.3389/fpubh.2023.1215462)
Supplement: Supplementary file 1 [file Table_1.DOCX]

**SUPPLEMENTAL TABLES**

**Supplementary Table 1. Women’s Empowerment Questionnaire**

*This interview should be conducted in privacy, where other members of the household or community cannot overhear or contribute answers. If you need to relocate at this time, ask the respondent if she minds asking others to step outside, or if you can go somewhere private. DO NOT read response options unless it specifically specifies to do so. Instead, enumerators should characterize responses based on the most applicable category/categories.*

**Role in Household Decision-making around production and income generation**

|  | | Question 1 | Question 2 | Question 3 | Question 4 | Question 5 |
| --- | --- | --- | --- | --- | --- | --- |
| “Now I’d like to ask you some questions about your participation in certain types of work activities and on making decisions on various aspects of household life” | | Did you yourself participate in [ACTIVITY] in the past 12 months (that is, during the last [one/two] cropping seasons), from [PRESENT MONTH] last year to [PRESENT MONTH] this year? | When decisions are made regarding [ACTIVITY], who is it that normally takes the decision?  **CIRCLE ALL APPLICABLE**  **IF THE RESPONSE IS SELF ONLY SKIP TO QUESTION G2.05** | How much input did you have in making decisions about [ACTIVITY]?  **USE DECISION CODES FOR G2.03/G2.05; IF NO DECISION MADE, ENTER 98 AND MOVE TO THE NEXT ACTIVITY** | To what extent do you feel you can make your own personal decisions regarding [ACTIVITY] if you want(ed) to? **CIRCLE ONE** | How much input did you have in decisions on the use of income generated from [ACTIVITY]  **USE CODES FOR G2.03/G2.05** |
| ACTIVITY CODE | ACTIVITY DESCRIPTION | G2.01 | G2.02 | G2.03 | G2.04 | G2.05 |
| **A** | Food crop farming: These are crops that are grown primarily for household food consumption | Yes….1  No…..2  If no, skip to activity B | SELF.........................................1 SPOUSE...................................2  OTHER HH MEMBER...............3  OTHER NON-HH MEMBER.....4  NOT APPLICABLE.................98 | NO INPUT OR INPUT IN FEW DECISIONS….01  INPUT INTO SOME DECISIONS..................02  INPUT INTO MOST OR ALL DECISIONS......03  NO DECISION MADE.............98 | NOT AT ALL ......... 1  SMALL EXTENT …... 2  MEDIUM EXTENT…… 3  TO A HIGH EXTENT. 4 | NO INPUT OR INPUT IN FEW DECISIONS…….01  INPUT INTO SOME DECISIONS......................02  INPUT INTO MOST OR ALL DECISIONS.........03  NO DECISION MADE..........98 |
| **B** | Cash crop farming: These are crops that are grown primarily for sale in the market |  |  |  |  |  |
| **C** | Livestock raising |  |  |  |  |  |
| **D** | Non-farm economic activities. This would include things like running a small business, self-employment, buy and sell |  |  |  |  |  |
| **E** | Wage and salary employment: This could be work that is paid for in cash or in-kind, including both agriculture and other wage work |  |  |  |  |  |
| **F** | Fishing or fishpond culture |  |  |  |  |  |
| **G** | Major household expenditures (such as a bicycles, land, boda boda) |  |  |  |  |  |
| **H** | Minor household expenditures (such as food for daily consumption or other household needs) |  |  |  |  |  |

**Supplementary Table 2: Proportion of women reporting participation in making decisions in agriculture and household activities**

| **Activity Description** | **Women participating in activity** | | **Women reporting making decisions^a^** | |
| --- | --- | --- | --- | --- |
|  | **n** | **%** | **n** | **%** |
| Food crop farming: Crops grown for household food consumption | 674 | 77.7 | 528 | 78.6 |
| Minor household expenditures (e.g., food for daily consumption or other household needs) | 602 | 69.4 | 508 | 84.4 |
| Cash crop farming: Crops that are grown primarily for sale in the market | 308 | 35.5 | 250 | 81.7 |
| Non-farm economic activities e.g., running a small business, self-employment, buy and sell | 252 | 29.0 | 212 | 84.1 |
| Major household expenditures (e.g., bicycles, land, boda boda) | 190 | 21.9 | 130 | 68.4 |
| Livestock raising | 162 | 18.7 | 132 | 81.5 |
| Wage and salary employment: Work paid for in cash or in-kind, incl agriculture and other wage work | 136 | 15.7 | 103 | 75.7 |
| Fishing or fishpond culture | 12 | 1.4 | 9 | 75.0 |

a/ Women were asked when decisions were made regarding activities they reported participation in who was it that normally took the decision? The table reports the proportion of women making decisions in agriculture activities that they reported they participated in - alone or jointly with other household members.
